# The Fecal Microbiota of Dogs Switching to a Raw Diet Only Partially Converges to That of Wolves

**DOI:** 10.3389/fmicb.2021.701439

**Published:** 2021-09-29

**Authors:** Jia Xu, Anne A. M. J. Becker, Yu Luo, Wenfu Zhang, Bingqian Ge, Chunqing Leng, Guyue Wang, Limin Ding, Jianmei Wang, Xiaoyu Fu, Geert P. J. Janssens

**Affiliations:** ^1^Department of Veterinary Medicine, Faculty of Agriculture, Jinhua Polytechnic, Jinhua, China; ^2^Department of Biomedical Sciences, Ross University School of Veterinary Medicine, Basseterre, Saint Kitts and Nevis; ^3^Department of Animal Nutrition, College of Animal Science and Technology, China Agricultural University, Beijing, China; ^4^Anbei Pet Food Inc., Beijing, China; ^5^Department of Nutrition, Genetics and Ethology, Faculty of Veterinary Medicine, Ghent University, Ghent, Belgium

**Keywords:** gut microbiome, canine, domestication, raw meat, wolves, nutrition

## Abstract

The genomic signature of dog domestication reveals adaptation to a starch-rich diet compared with their ancestor wolves. Diet is a key element to shape gut microbial populations in a direct way as well as through coevolution with the host. We investigated the dynamics in the gut microbiota of dogs when shifting from a starch-rich, processed kibble diet to a nature-like raw meat diet, using wolves as a wild reference. Six healthy wolves from a local zoo and six healthy American Staffordshire Terriers were included. Dogs were fed the same commercial kibble diet for at least 3 months before sampling at day 0 (DC), and then switched to a raw meat diet (the same diet as the wolves) for 28 days. Samples from the dogs were collected at day 1 (DR1), week 1 (DR7), 2 (DR14), 3 (DR21), and 4 (DR28). The data showed that the microbial population of dogs switched from kibble diet to raw diet shifts the gut microbiota closer to that of wolves, yet still showing distinct differences. At phylum level, raw meat consumption increased the relative abundance of Fusobacteria and Bacteroidetes at DR1, DR7, DR14, and DR21 (*q* < 0.05) compared with DC, whereas no differences in these two phyla were observed between DC and DR28. At genus level, *Faecalibacterium*, *Catenibacterium*, *Allisonella*, and *Megamonas* were significantly lower in dogs consuming the raw diet from the first week onward and in wolves compared with dogs on the kibble diet. Linear discriminant analysis effect size (LEfSe) showed a higher abundance of *Stenotrophomonas*, *Faecalibacterium*, *Megamonas*, and *Lactobacillus* in dogs fed kibble diet compared with dogs fed raw diet for 28 days and wolves. In addition, wolves had greater unidentified Lachnospiraceae compared with dogs irrespective of the diets. These results suggested that carbohydrate-fermenting bacteria give way to protein fermenters when the diet is shifted from kibble to raw diet. In conclusion, some microbial phyla, families, and genera in dogs showed only temporary change upon dietary shift, whereas some microbial groups moved toward the microbial profile of wolves. These findings open the discussion on the extent of coevolution of the core microbiota of dogs throughout domestication.

## Introduction

The concept of “hologenome” has been proposed to imply the genetic role of both the host and its associated microorganisms throughout evolution (Zilber-Rosenberg and Rosenberg, 2008). Dogs (*Canis familiaris*) are possibly the first domesticated species, having diverged from the gray wolf (*Canis lupus*) more than 15,000 years ago (Freedman et al., 2016). Wolves can be considered true carnivores in their nature with vegetal matter being a minor to negligible component of their overall diet, whereas dogs have adapted to a more flexible anthropogenic diet including starchy food (Bosch et al., 2015). Compared with their wild ancestors, dogs have become more adapted to a starch-rich diet as demonstrated by the pancreatic α-amylase 2B (AMY2B) copy number expansion in the genome of the dog (Axelsson et al., 2013; Arendt et al., 2016; Ollivier et al., 2016; Reiter et al., 2016).

Likely, domestication and its associated change in feeding habits not only affect the canine genome but also shape their associated gut bacterial populations. Compared with wolves feeding on raw carcasses, dogs feeding on human food leftovers and commercial pet foods hold indeed more amylolytic gut bacteria such as *Ruminococcaceae*, *Desulfuromonadaceae*, and *Faecalibacterium* (Lyu et al., 2018). Likewise, the gut microbiota of another strict carnivore, the cheetah, was distinctly different in composition from the gut microbiota of domestic cats fed starch-rich, processed diets (Becker et al., 2014).

Many studies have also evaluated the impact of raw-meat based diets and bones and raw food (BARF) diets on the fecal microbiota in dogs (Bermingham et al., 2017; Kim et al., 2017; Sandri et al., 2017; Schmidt et al., 2018). The increased abundance of *Clostridium perfringens* and *Fusobacterium varium* is associated with raw diet consumption (Kim et al., 2017; Schmidt et al., 2018). Both the bacterial composition and metabolic repertoire of the canine gut microbiota have evolved to adapt to high digestible protein or high carbohydrate intake (Alessandri et al., 2020; Huang et al., 2020). Most of these studies assess microbial changes between dogs on different diets at a single time point, but few studies have evaluated such microbial changes in the light of coevolution of gut microbial communities following domestication of the dog. In this study, we used wolves consuming a raw meat-based diet as a reference to investigate the diversity and dynamics of the gut microbial populations in dogs shifting from a starch-rich, processed kibble diet to a raw meat-based diet identical to that of the wolves.

## Materials and Methods

### Ethics Statement

The study was approved by the Ethical Committee of Jinhua Polytechnic (NXY2018/01). Written informed consent was obtained from the owners for the participation of their animals in this study.

### Animals and Study Design

Six healthy adult wolves (W) from Yancheng Wild Zoo with no clinical signs, such as, but not limited to, vomiting and diarrhea, were included. The wolves were living in a group with an open enclosure. During the feeding and sampling period, the wolves were housed individually in a 3 m × 5 m enclosure. Six healthy American Staffordshire Terriers from Anbei Pet Food Inc. were also included. Dogs were housed individually in 1.5 m × 3 m houses with access to a 20 m × 30 m outdoor enclosure. Both dogs and wolves received no medication for at least 6 months prior to sampling. The metadata of the animals are shown in Supplementary Table 1.

Each wolf was fed 1.25 kg of raw chicken meat and bone plus 0.25 kg of raw chicken without feathers and organs once daily except a fasting day on Friday and only half of the daily amount fed on Monday. Vitamin and mineral supplements were provided monthly during 1 week. Dogs were fed the same kibble diet for at least 3 months before sampling at day 0, and then switched to the same raw diet as that of the wolves for 28 days. The dogs were fed twice a day in the morning and afternoon; the amount was given to maintain the body weight. The chemical composition of the kibble diet is shown in Supplementary Table 2.

### Sampling

Fresh fecal samples from six wolves (W) were collected after diet consumption. Fresh fecal samples from six dogs were collected at day 0 (dogs fed commercial diet, DC), day 1 (dogs fed raw diet at day 1, DR1), day 7 (dogs fed raw diet at week 1, DR7), day 14 (dogs fed raw diet at week 2, DR14), day 21 (dogs fed raw diet at week 3, DR21), and day 28 (dogs fed raw diet at week 4, DR28). All fecal samples were flash frozen in dry ice and stored at −80°C until analysis. Samples were shipped to Novogene Co., Ltd. (Beijing) for further analysis.

### Extraction of Genomic DNA and Amplicon Generation

Total genomic DNA from samples was extracted using the CTAB/SDS method (Zhang et al., 2006). DNA concentration and purity were monitored on 1% agarose gels. According to the concentration, DNA was diluted to 1 ng/μl using sterile water. DNA samples were stored at −80°C until further analyses. Amplification of the V3–V4 region of the 16S rRNA gene was performed in 30-μl reactions with 15 μl of Phusion^®^ High-Fidelity PCR Master Mix (New England Biolabs), 0.2 μM of forward (341F: CCTAYGGGRBGCASCAG) and reverse (806R: GGACTACNNGGGTATCTAAT) primers, and 10 ng of template DNA. Thermal cycling consisted of initial denaturation at 98°C for 1 min, followed by 30 cycles of denaturation at 98°C for 10 s, annealing at 50°C for 30 s, elongation at 72°C for 30 s, and finally 72°C for 5 min. PCR products were mixed in equidensity ratios and purified with GeneJET^TM^ Gel Extraction Kit (Thermo Fisher Scientific).

### Library Preparation and Sequencing

Sequencing libraries were generated using Ion Plus Fragment Library Kit 48 rxns (Thermo Fisher Scientific) following the recommendations of the manufacturer. The library quality was assessed on the Qubit@ 2.0 Fluorometer (Thermo Fisher Scientific). At last, the library was sequenced on an Ion S5^TM^ XL platform and 400-/600-bp single-end reads were generated.

### Data Analysis

Quality filtering of the raw single-end reads was performed under specific filtering conditions to obtain the high-quality clean reads according to the Cutadapt (Martin, 2011) quality controlled process. The reads were compared with the reference database (Silva database) (Quast et al., 2013) using UCHIME algorithm (UCHIME Algorithm) (Edgar et al., 2011) to detect chimera sequences, which were subsequently removed (Haas et al., 2011).

Sequence analysis was performed by UPARSE software (UPARSE v7.0.1001) (Edgar, 2013). Sequences with ≥97% similarity were assigned to the same operational taxonomic units (OTUs). A representative sequence for each OTU was selected for further annotation.

Alpha diversity metrices including Observed-species, Chao1, Shannon, and Good’s coverage were calculated with QIIME (Version 1.9.1), and rarefaction curve was analyzed using R software (Version 2.15.3) (Supplementary Figure 1). Data were normalized to the lowest reads/sample to decrease bias caused by non-uniform sequencing depth. Beta diversity was evaluated by weighted and unweighted UniFrac distance matrices and visualized using principal coordinate analysis (PCoA) plots. Metastats was used to identify differentially abundant genera between the different groups (White et al., 2009). The resulting *p*-values were adjusted for multiple comparisons using the false discovery rate (FDR), and an adjusted *q* < 0.05 was considered statistically significant (Benjamini and Hochberg, 1995).

Singletons were removed for taxonomic composition and differential abundance analyses, and OTUs assigned at the phyla, family, and genus levels whose relative median abundance was <1% were not presented. All the relative abundance data were expressed as median relative abundances. Intergroup differences of the microbiota were also calculated by permutational analysis of variance (PERMANOVA, adonis function in the vegan package).

Linear discriminant analysis effect size (LEfSe) was used to elucidate bacterial taxa (16S rRNA genes), which are different between groups. LEfSe was used in the Galaxy workflow framework with the parameters set at α = 0.01, LDA score = 4.0.

Tax4Fun functional prediction was achieved by the nearest neighbor method based on the minimum 16S rRNA gene sequence similarity (Aßhauer et al., 2015). A correlation matrix was established by extracting the KEGG database prokaryotic whole genome 16S rRNA gene sequence and aligning it to the SILVA SSU Ref NR database using BLASTN algorithm (BLAST Bitscore >1,500). The SILVA 132 database function annotation was implemented via mapping the prokaryotic whole genome functional information of the KEGG database annotated by UProC and PAUDA to the SILVA 132 database. The sequenced samples were clustered out of the OTU using the SILVA 132 database sequence as a reference sequence to obtain functional annotation information.

## Results

### Characterization and Comparison of Gut Microbiota Between Dogs and Wolves

**FIGURE 1:**
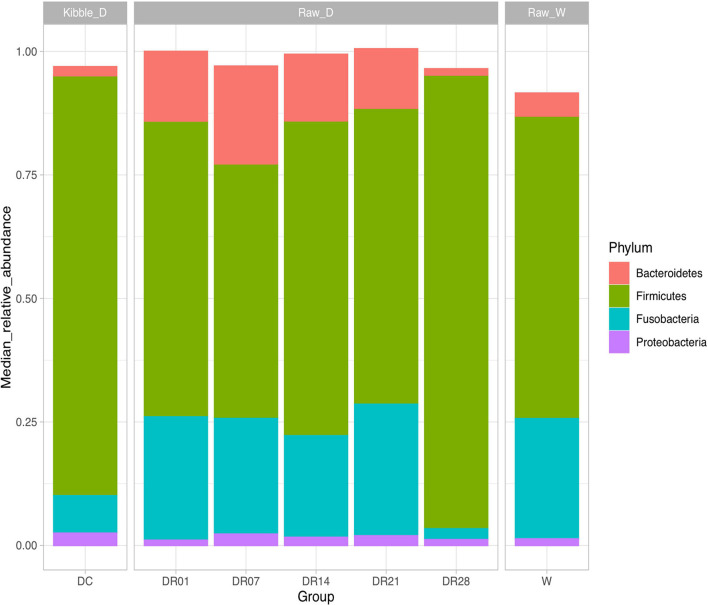
Top microbial phyla observed (relative median abundance was >1%) in feces of raw-fed wolves and dogs on a diet shift from a processed kibble diet to a raw diet.

A total of 3,354,245 16S rRNA gene-based amplicon sequences were obtained, with an average of 79,863 reads (range = 71,099–80,247) per fecal sample, from which 44,688 reads per sample were randomly subsampled to normalize sequence numbers. The number of obtained operational taxonomic unites (OTUs) for DC, DR1, DR7, DR14, DR21, and DR28 were 217, 203, 188, 173, 185, 187, and 189, respectively, and the Good’s coverage of the clone libraries was 99.96 ± 0.05% (Supplementary Table 3). The most abundant phyla in both dogs and wolves were Firmicutes and Fusobacteria, followed by Bacteroidetes and Proteobacteria (Figure 1). Dogs and wolves shared 133 OTUs. The OTU list is shown in Supplementary Table 4.

*Peptoclostridium* (26.90%), *Lactobacillus* (17.13%), *Megamonas* (9.37%), and *Faecalibacterium* (8.59%) were the four most abundant genera in dogs fed the commercial diet; *Fusobacterium* (24.21%) and *Peptoclostridium* (17.30%) were the most abundant genera in wolves (Supplementary Figure 2). However, large individual differences still existed (Supplementary Figure 3).

**FIGURE 2:**
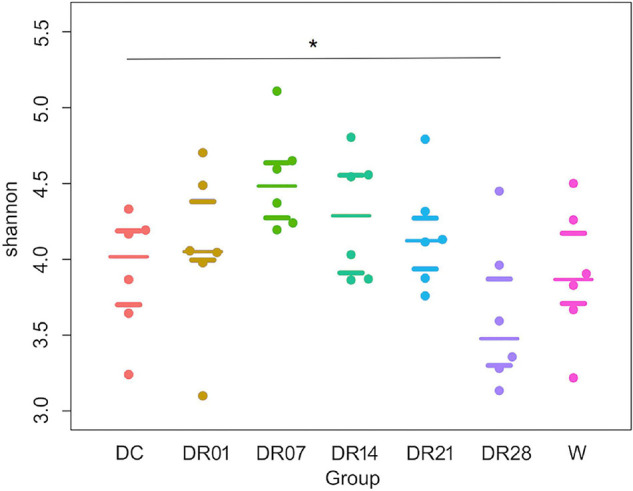
Shannon index with median and interquartile ranges for faces samples of raw-fed wolves and dogs on a diet shift from a processed kibble diet to a raw diet (an asterisk means that *q* < 0.05).

The diversity assessed by the Shannon index was significantly higher in dogs fed kibble diet compared with dogs fed raw diet for 28 days (*q* < 0.05, Figure 2). No differences were observed on other index of microbial richness and diversity among dogs that were fed kibble diet, dogs fed raw diet, and wolves (*q* > 0.05).

**FIGURE 3:**
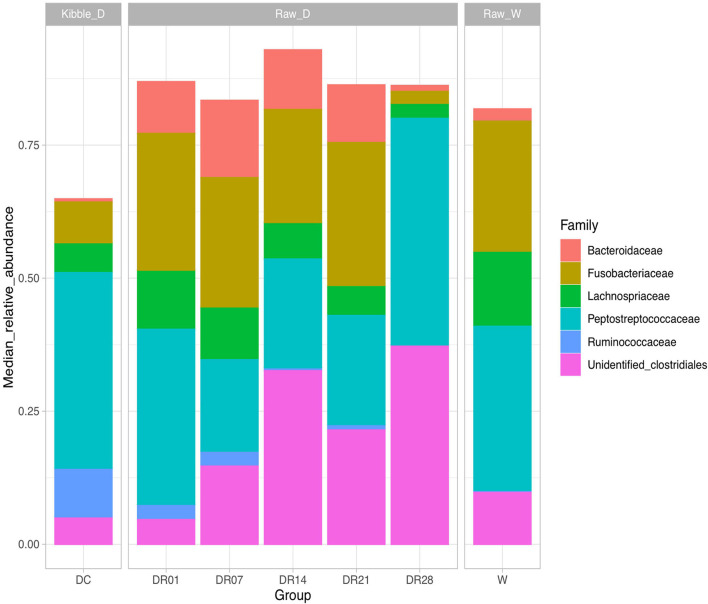
Top microbial families observed (relative median abundance was >1%) in feces of raw-fed wolves and dogs on a diet shift from a processed kibble diet to a raw diet.

Gut microbiota in dogs fed commercial diet and raw diet at days 7, 14, and 28 were significantly different from wolves (PERMANOVA, *R*^2^ = 0.32, 0.33, 0.27, and 0.38, respectively, *p* < 0.05). Many distinct differences were observed between dogs and wolves in relative abundance at both family (Figure 3) and genus levels (Supplementary Figure 2). At family level (Supplementary Figure 4), the relative abundance of Lachnospiraceae was significantly higher (14.14 vs. 5.61%), whereas Ruminococcaceae was significantly lower (0.41 vs. 9.32%) in wolves than in dogs fed the commercial diet (*q* < 0.05). At genus level, the relative abundance unidentified Lachnospiraceae was significantly higher in wolves compared with dogs fed kibbles and raw diet for 28 days (Supplementary Figure 5); *Acidothermus*, *Methylovirgula*, and *Candidatus Solibacter* were significantly higher in wolves compared with dogs irrespective of the diet (*q* < 0.05); however, the relative abundance of these genera were <0.01% (data not shown). Based on LEfSe analysis, a higher abundance of *Stenotrophomonas*, *Faecalibacterium*, *Megamonas*, and *Lactobacillus* and a lower abundance of unidentified Lachnospiraceae were found in dogs fed kibble diet compared with wolves (Supplementary Figure 6). In addition, the relative abundance of *Fusobacterium*, *Romboutsia*, and unidentified Lachnospiraceae were higher, whereas *Anaerobiospirillum* and unidentified Clostridiales were lower in wolves compared with dogs fed raw diet for 28 days (Supplementary Figure 6).

**FIGURE 4:**
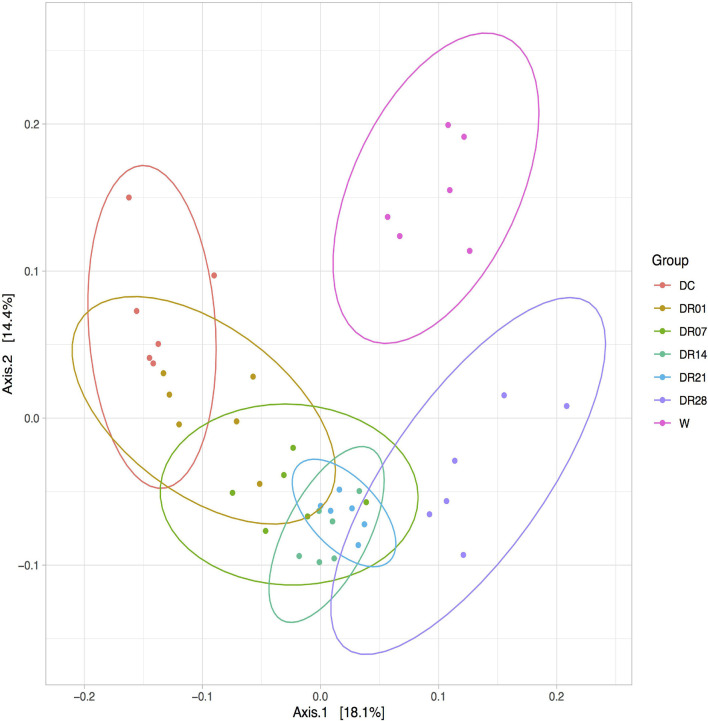
Principal coordinate analysis (PCoA) analysis of unweighted UniFrac distances of 16S rRNA genes in feces of raw-fed wolves and dogs on a diet shift from a processed kibble diet to a raw diet.

Principal coordinate analysis plots based on the unweighted UniFrac distance metric indicated separation of samples between dogs and wolves. The clustering of the fecal microbiota of dogs fed raw diet especially after 4 weeks was closer to wolves compared with dogs fed kibble diets (Figure 4).

### Gut Microbiota Dynamics in Dogs Shifting From Kibble to Raw Diet

Changing from kibble to raw diet significantly modified gut microbiota in dogs. The gut microbiota of dogs fed commercial diet was significantly different from dogs fed raw diet at days 1, 7, 14, 21, and 28 (PERMANOVA, *R*^2^ = 0.33, 0.51, 0.54, 0.51, 0.53, respectively, *p* < 0.01).

**FIGURE 5:**
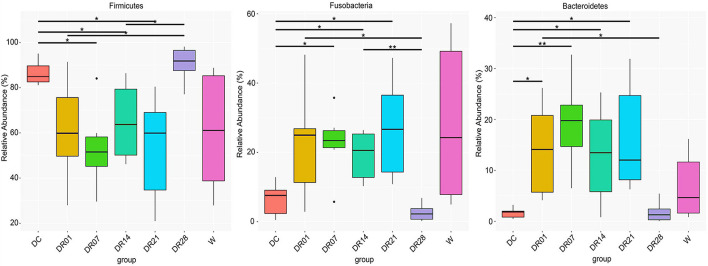
The relative abundance of fecal microbiota at phylum level in feces of raw-fed wolves and dogs on a diet shift from a processed kibble diet to a raw diet (an asterisk means that *q* < 0.05; a double asterisk means that *q* < 0.01).

The change of the diet from commercial kibbles to raw meat in dogs promoted many notable differences in relative abundance at phylum (Figure 5), family (Supplementary Figure 4), and genus levels (Supplementary Figure 5). At phylum level, raw meat consumption increased the relative abundance of Fusobacteria from the first week and Bacteroidetes from the first day until the third week (*q* < 0.05) compared with dogs on the commercial diet. However, no differences in the relative abundance of Fusobacteria and Bacteroidetes were observed between the kibble diet and raw diet at the final sampling on day 28. On the contrary, the relative abundance of Firmicutes was decreased after the first week change of the diet until the third week. That difference with the kibble diet was no longer observed on week 4 (*q* < 0.05).

At family level (Supplementary Figure 4), the relative abundance of Ruminococcaceae and Veillonellaceae were significantly higher in dogs fed kibble diet than dogs fed raw diet (DR14, DR21, and DR28). The relative abundance of Bacteroidaceae and Fusobacteriaceae was transiently increased in DR7 and DR21 in dogs fed raw diet compared with kibble diet. The relative abundance of unidentified Clostridiales was much higher in dogs fed raw diet (DR14, DR21, and DR28) compared with DC.

At genus level (Supplementary Figure 5), the relative abundance of *Bacteroides* in DR7 and DR21 was higher than in DC (*q* < 0.05), and *Fusobacterium* in DR7, DR14, and DR21 was higher than in DC (*q* < 0.05). However, there were no differences in the relative abundance of *Bacteroides* and *Fusobacterium* between DC and DR28. In addition, compared with DC, the relative abundance of *Faecalibacterium*, *Megamonas*, *Romboutsia*, *Allisonella* (<0.1%), and *Catenibacterium* (<0.1%) in dogs fed the raw diet (DR7, DR14, DR21, and DR28), and in wolves, it was significantly lower compared with DC. The abundance of *Sarcina* and *Turicibacter* were also lower in DR7 (*q* < 0.05, *q* < 0.05), DR14 (*q* = 0.053, *q* = 0.053), DR21 (*q* < 0.05, *q* < 0.05), DR28 (*q* = 0.058, *q* = 0.052), and W (*q* < 0.05, *q* < 0.05) compared with DC, respectively. *Bifidobacterium* was significantly decreased in DR28 (*q* < 0.05). In addition, the relative abundance of unidentified Clostridiales was increased in dogs fed raw meat diet (DR14, DR21, and DR28) compared with dogs fed kibble diet (Supplementary Figure 5); the relative abundances of *Collinsella*, *Enterococcus*, *Slackia*, *Candidatus Stoquefichus*, *Faecalitalea*, *Lactococcus*, and *Candidatus Saccharimonas* were significantly increased in DR28 (*q* < 0.05) compared with dogs on kibble diet (<0.1%, data not shown). Furthermore, based on LEfSe analysis, a higher abundance of *Stenotrophomonas*, *Romboutsia*, *Faecalibacterium*, *Megamonas*, and *Lactobacillus* and a lower abundance of unidentified Clostridiales, *Paeniclostridium* were detected in dogs fed kibble diet compared with dogs fed raw diet for 28 days (Supplementary Figure 6).

### Predicted Functional Composition of Fecal Microbial Communities in Dogs and Wolves

Although significant changes on gut microbial composition were observed between dogs and wolves, much less variation was observed for predicted functional composition (Supplementary Figure 7). At the first level of the functional categories, which include metabolism, genetic information processing, environmental information processing, cellular processes, and so on, no difference was observed between DC and W. Changing diet from kibble to raw induced functional changes from day 1 to the first 3 weeks. At the fourth week, only one category was different between DC and DR28, which is cellular processes (Supplementary Figure 8); at the second highest level of the functional categories, similar changes were observed (Supplementary Figure 9). Interestingly, at this level, carbohydrate metabolism was significantly higher in dogs fed raw diets and wolves compared with dogs fed kibble diets (*p* < 0.01). Principal component analysis (PCA) was performed on predicted metagenomes KEGG second level. As shown on PC1 (71.93%), we could see that the DC and DR28 clustered closer to each other on the horizontal axis (Supplementary Figure 10).

## Discussion

Our study has shown that when dogs shift from a starch-rich, processed kibble to a more nature-like raw-meat diet, the gut microbial populations have three patterns of changes within 4 weeks after diet alteration: (1) Some microbial populations changed when switching the diet but tended to stabilize and return to the starting point after 4 weeks. (2) Some microbial populations changed toward that of wolves after fed the raw diet. (3) Differences remain between dogs and wolves regardless of the diet. Therefore, our study may suggest that the actual diet as well as phylogeny determine the composition of gut microbiota, which is in accordance with previous studies across species (Ley et al., 2008; Youngblut et al., 2019). However, to what extent of the diet or coevolution of the core microbiota of dogs throughout domestication has affected those difference remains unclear.

As practical limitations to our study, limited numbers of the animals and differences in feeding regime existed, including the feeding frequency and fasting. In addition, the raw diet of the zoo wolves that was also used in the diet shift of the dogs still differed from the eating of (fresh and cached) carcass parts in the wild. Nevertheless, this diet will already match the natural diet much closer than a processed diet with very little physical structure and fibrous parts. The importance of such fibrous matter in a carnivorous diet was demonstrated by Depauw et al. (2013).

In agreement with other studies, Firmicutes were the most dominant phylum in the dog fecal samples (Li et al., 2017; Vilson et al., 2018). Many studies have reported Bacteroidetes as the second dominant phylum (Beloshapka et al., 2013; Li et al., 2017), but in our study, Fusobacteria were the second dominant phylum. Although 16s rRNA 454-pyrosequencing were used in previous mentioned studies, different regions were amplified. Yet, a plausible explanation for the discordance with our study is that the diets in these studies still contained considerable amounts of plant-based ingredients. One study found that Actinobacteria was the second most dominant phylum in German Shepherd (Vilson et al., 2018). In another study, Fusobacteria were found to be higher in the Maltese than in the Miniature Schnauzer (Reddy et al., 2019). It must be acknowledged that these studies not only differ in breed but also in diet history. Since our study suggests that some microbial groups may take a long time to respond to dietary changes, diet effects on the dominant phyla cannot be ruled out.

The transient rise of Fusobacteria (Fusobacteriaceae) and Bacteroidetes (Bacteroidaceae) and the transient decrease of Firmicutes (Erysipelotrichaceae) point to a microbial profile due to the shifting diet *per se* rather than the influence of the raw diet. The microbial population changes seem to occur due to the diet change *per se* and may, thus, not reflect the fully adapted microbiome. It is important to take into account that in case of sampling when the microbiota is still in a transitory phase, these transient microbial changes may be erroneously considered as the adapted microbial populations. Nevertheless, some bacterial groups reacted immediately or at least consistently to the diet shift. Their function can be related to diet composition and what can be expected to end up as substrate in the hindgut.

Changing the dogs’ diet from kibble to a raw diet may force carbohydrate fermenting bacteria to give way to protein fermenters and move toward the profile of wolves. The genera *Romboutsia*, *Faecalibacterium*, *Catenibacterium*, *Megamonas*, and *Allisonella* quickly dropped when dogs fed kibble switched to the raw diet and did not rise until the end of the study. Other studies also found the reduction in *Faecalibacterium* when fed a bones and raw food (BARF) diet (Schmidt et al., 2018; Alessandri et al., 2019). The genera *Sarcina* and *Turicibacter* exhibited the same tendency but did show a small increasing trend on the fourth week. The decreases in *Faecalibacterium* and *Turicibacter* have been associated with IBD and acute diarrhea in dogs (Suchodolski et al., 2012; Rossi et al., 2014; Minamoto et al., 2015). Studies also found that *Catenibacterium*, *Megamonas*, and *Allisonella* were more prominent in a processed diet compared with a BARF diet in dogs and cats (Schmidt et al., 2018; Butowski et al., 2019). These genera are all able to ferment carbohydrates to produce short-chain fatty acids (SCFA; Kieler et al., 2017; Butowski et al., 2019; Che et al., 2019). In addition, *Stenotrophomonas* that has a higher abundance in dogs fed kibble vs. raw diet also had a positive correlation to the relative amount of both acetate and propionate (Nilsen et al., 2020). Interestingly, adding inulin to a BARF diet increased the abundance of *Megamonas* (Butowski et al., 2019).

Except *Romboutsia*, the relative abundance of the genera mentioned above (i.e., *Faecalibacterium*, *Catenibacterium*, *Megamonas*, and *Allisonella*) in dogs fed kibble diet also significantly differed from that of wolves. The genomic analysis of the genus *Romboutsia* revealed its broad range of metabolic capabilities with respect to carbohydrate utilization, fermentation of single amino acids, anaerobic respiration, and metabolic end products (Gerritsen et al., 2019). Based on our results, *Romboutsia* might play a more important role in carbohydrate utilization in the hindgut of dogs. All those genera belong to Firmicutes; the relative abundance of this phylum was decreased for the first 3 weeks after dietary change. Kibble diets are higher in carbohydrates and lower in protein and fat compared with raw diets. This matches the observed shift in bacterial populations away from carbohydrate fermenters when on the raw diet.

Different strains of *Lactobacillus* and *Bifidobacteria* genera possess significant and widely acknowledged health-promoting properties in various mammalian omnivores, including humans, mice, and pigs (Vlasova et al., 2017). In this study, *Lactobacillus* was one of the most dominant genera in dogs fed kibble diet, and its abundance was also significantly higher in dogs fed kibble diet compared with dogs fed raw diet for 28 days and wolves according to LEfSe analysis. Some stains of *Lactobacillus* such as *Lactobacillus reuteri* and *Lactobacillus johnsonii* have been proposed as potential probiotic (Kumar et al., 2017). However, a lingering question is whether such probiotics would be effective in raw-fed dogs and by extension in all natural-fed carnivorous mammals since an increasing amount of studies point to a low natural abundance of pathogens that may thrive on carbohydrates (e.g., cheetahs) (Becker et al., 2014), hence, reducing the relevance of Lactobacillaceae probiotics. Similarly, after the 4 weeks of raw feeding, the relative abundance of *Bifidobacteria* was much lower compared with the kibble diet. Supplementation of *Bifidobacteria* strains has shown positive effects on dogs with acute diarrhea but again in dogs fed starchy processed diets (Kelley et al., 2009). An increase in Lactobacillaceae and Bifidobacteriaceae, thus, may not always be interpreted as beneficial on all diet types as the abundance of these bacteria were also lower in cheetahs on a natural diet (Becker et al., 2015).

Unidentified Clostridiales continually increased in dogs fed raw meat diet compared with dogs fed kibble diet; one of the contributing factors could be *Clostridium perfringens.* An increase in *C. perfringens* was also found in dogs fed a high protein diet or BARF diet (Kim et al., 2017; Li et al., 2017; Schmidt et al., 2018). *C. perfringens* was found to be present in a high abundance in healthy dogs as well as other carnivorous species and is considered a common commensal in carnivores (Muegge, 2013). A study found that *C. perfringens* was associated with the butyrate kinase gene, a functional gene in butyrate production (Vital et al., 2014). It should be noted that the relative abundance of unidentified Clostridiales was lower in wolves compared with dogs fed raw diet for 28 days. This could be due to the fact that gut microbiota of dogs have not reached a stable status after 28 days of feeding. Moreover, wolves had greater unidentified Lachnospiraceae; this is in accordance with a previous study that found cat fed chicken also had greater unidentified Lachnospiraceae compared with cat fed an extruded chicken-based diet (Kerr et al., 2014). Therefore, the changes in these bacteria would be beneficial for protein fermentation.

Using 16S rRNA genes for microbial function prediction, we observed the dynamic changes in microbial functional redundancy. More predicted differences of microbial function were exhibited when the microbial populations were not stable (week 1–3) than when they were more stable (week 4). Carbohydrate metabolism was significantly higher in wolves and dogs on the raw diet than in dogs consuming kibble diet. It may be confusing that microbial function predictions of a diet low in carbohydrates shows increased “carbohydrate metabolism.” Yet, most amino acids can easily feed into the carbohydrate metabolism pathways that are described by Rowland et al. (2018). The microbial use of amino acids as an energy source is, therefore, the most plausible explanation for this predicted feature, as demonstrated by Xu et al. (2017). For example, *C. perfringens* is associated with high dietary protein and possesses the ability to produce butyrate (Louis et al., 2004). This is supported by an isotope study that found gut microbiota were associated with more essential amino acid production when the diet was rich in carbohydrate instead of rich in protein (Newsome et al., 2020). Still, we need to appreciate that the result of microbial function was predicted in this study; 16S rRNA was used instead of genomic DNA. Multiomic techniques including metagenomic, proteomics, and metabolomics would provide more accurate results on microbial function.

In conclusion, the dynamic change in the gut microbiota of the dog after a diet shift from a processed kibble diet to a raw diet increases protein fermenters and decreases the abundance of bacteria for carbohydrate fermentation, showing an increasing resemblance with the microbial profile of wolves. Yet, transient changes also occurred, and caution is needed to avoid misinterpretation. The bacteria groups still show distinct differences between wolves and dogs after 4 weeks on the same diet. This may suggest, but not yet prove, a divergence from wolves during the dog domestication; long-term feeding is needed to further explore the impact of diet and phylogeny on canine gut microbiota.

## Data Availability Statement

The datasets presented in this study can be found in online repositories. The names of the repository/repositories and accession number(s) can be found below: SRA Database, Accession Number: PRJNA729861.

## Ethics Statement

The study was approved by the Ethical Committee of Jinhua Polytechnic (NXY2018/01). Written informed consent was obtained from the owners for the participation of their animals in this study.

## Author Contributions

JX and GJ conceived and designed the experiment. JX, YL, WZ, JW, LD, and XF performed the experiment. JX, WZ, BG, CL, and GW carried out the analyses. JX, AB, and GJ performed the statistical analysis, and drafted and amended the manuscript. All authors contributed to the article and approved the final manuscript.

## Conflict of Interest

XF was employed by the company Anbei Pet Food Inc., Beijing, China. The remaining authors declare that the research was conducted in the absence of any commercial or financial relationships that could be construed as a potential conflict of interest.

## Publisher’s Note

All claims expressed in this article are solely those of the authors and do not necessarily represent those of their affiliated organizations, or those of the publisher, the editors and the reviewers. Any product that may be evaluated in this article, or claim that may be made by its manufacturer, is not guaranteed or endorsed by the publisher.

## Supplementary Material

Supplementary Figure 1 Rarefaction curves for all the samples.

Supplementary Figure 2 Top microbial genera observed (relative median abundance was >1%) in feces of raw-fed wolves and dogs on a diet shift from a processed kibble diet to a raw diet.

Supplementary Figure 3 Number of operational taxonomic units (OTUs) in different groups. Each box plot represents median, interquartile range, minimum, and maximum values.

Supplementary Figure 4 The relative abundance of fecal microbiota at family level in feces of raw-fed wolves and dogs on a diet shift from a processed kibble diet to a raw diet (an asterisk means that *q* < 0.05; a double asterisk means that *q* < 0.01).

Supplementary Figure 5 The relative abundance of fecal microbiota at genus level in feces of raw-fed wolves and dogs on a diet shift from a processed kibble diet to a raw diet (an asterisk means that *q* < 0.05; a double asterisk means that *q* < 0.01).

Supplementary Figure 6 Linear discriminant analysis effect size (LEfSe) analysis of 16S rRNA gene sequences. Only LDA values >4 are shown in **(A–C)**.

Supplementary Figure 7 Predicted functional composition of metagenomes based on 16S rRNA gene sequencing data.

Supplementary Figure 8 The differences of the functional categories at the first level between dogs fed kibble diet and raw diet at different time points. **(a)** Dogs fed kibble diet vs. dogs fed raw diet for 1 day. **(b)** Dogs fed kibble diet vs. dogs fed raw diet for 7 days. **(c)** Dogs fed kibble diet vs. dogs fed raw diet for 14 days. **(d)** Dogs fed kibble diet vs. dogs fed raw diet for 21 days. **(e)** Dogs fed kibble diet vs. dogs fed raw diet for 28 days.

Supplementary Figure 9 The differences of the functional categories at the second level between dogs fed commercial diet and raw diet at different time points. **(a)** Dogs fed kibble diet vs. dogs fed raw diet for 1 day. **(b)** Dogs fed kibble diet vs. dogs fed raw diet for 7 days. **(c)** Dogs fed kibble diet vs. dogs fed raw diet for 14 days. **(d)** Dogs fed kibble diet vs. dogs fed raw diet for 21 days. **(e)** Dogs fed kibble diet vs. dogs fed raw diet for 28 days. **(f)** Dogs fed kibble diet vs. wolves. **(g)** Dogs fed raw diet for 28 days vs. wolves.

Supplementary Figure 10 Principal component analysis of the predicted gene function in raw-fed wolves and dogs on a diet shift from a processed kibble diet to a raw diet.
