# Supplementary figures and images for: The Fecal Microbiota of Dogs Switching to a Raw Diet Only Partially Converges to That of Wolves
Source: Front Microbiol. 2021 Sep 29;12:701439. doi: 10.3389/fmicb.2021.701439 (PMC8511826; doi:10.3389/fmicb.2021.701439)

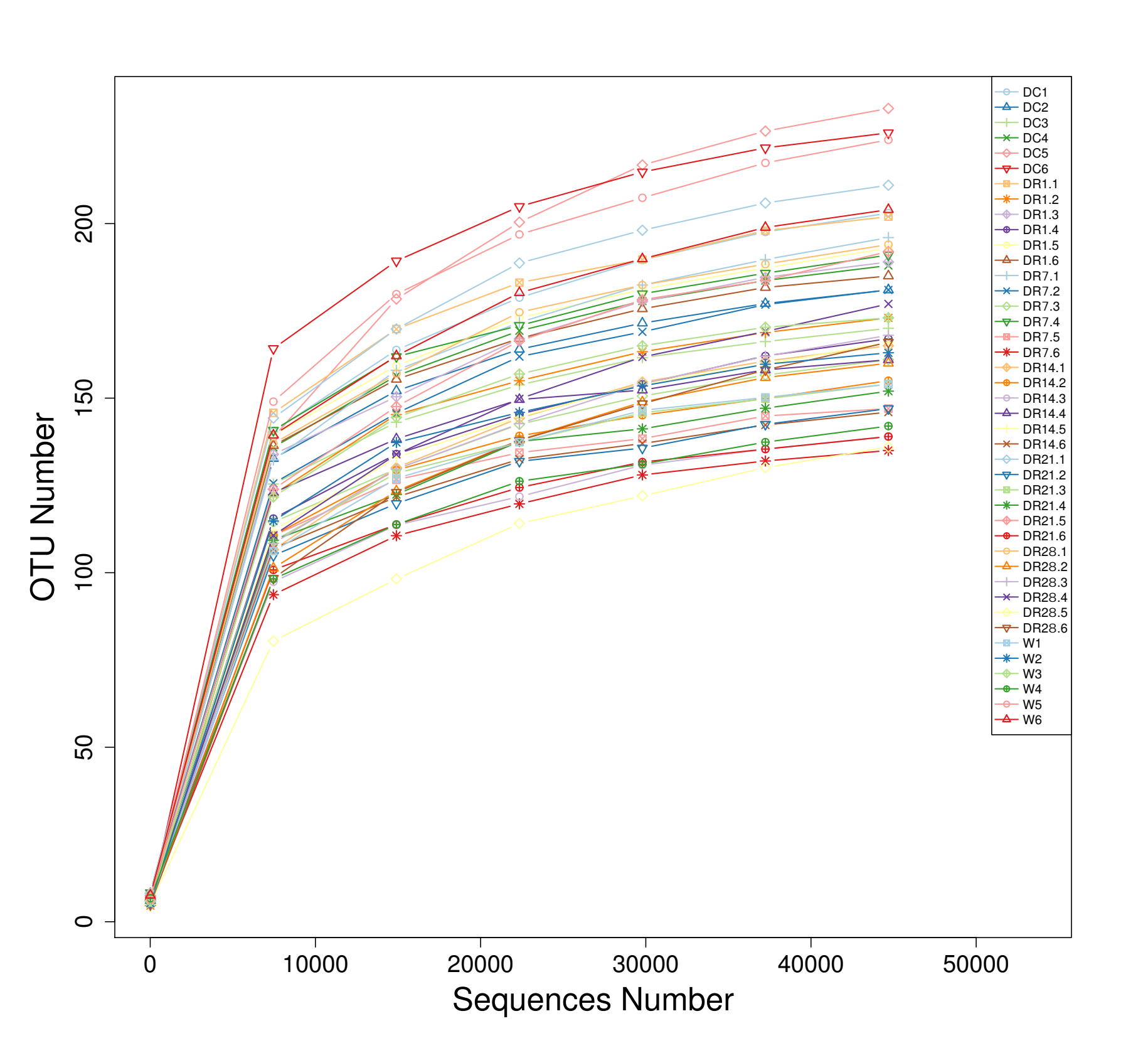

Supplement: Supplementary Figure 1 — Rarefaction curves for all the samples. [file Image_1.PNG]

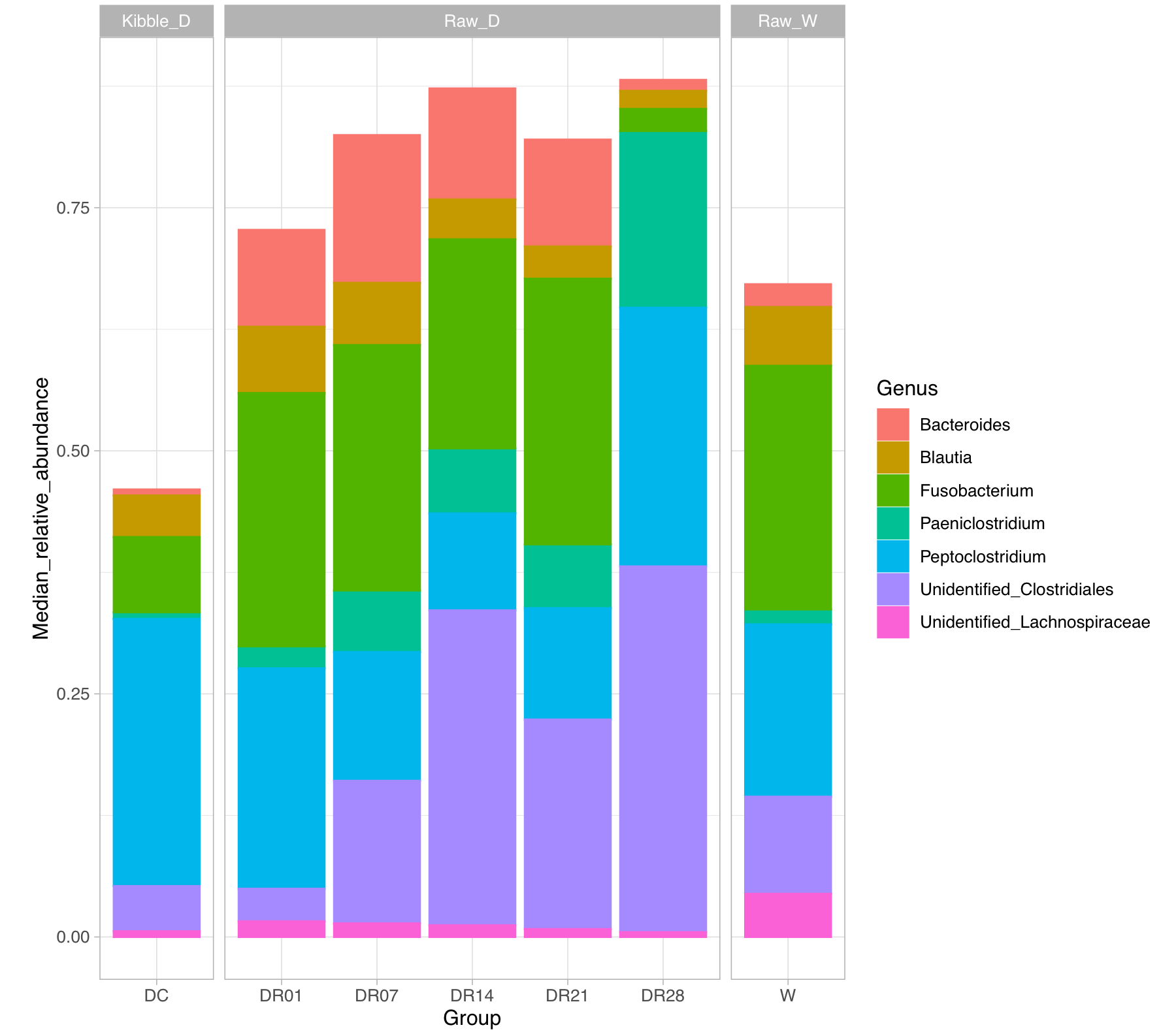

Supplement: Supplementary Figure 2 — Top microbial genera observed (relative median abundance was >1%) in feces of raw-fed wolves and dogs on a diet shift from a processed kibble diet to a raw diet. [file Image_2.PNG]

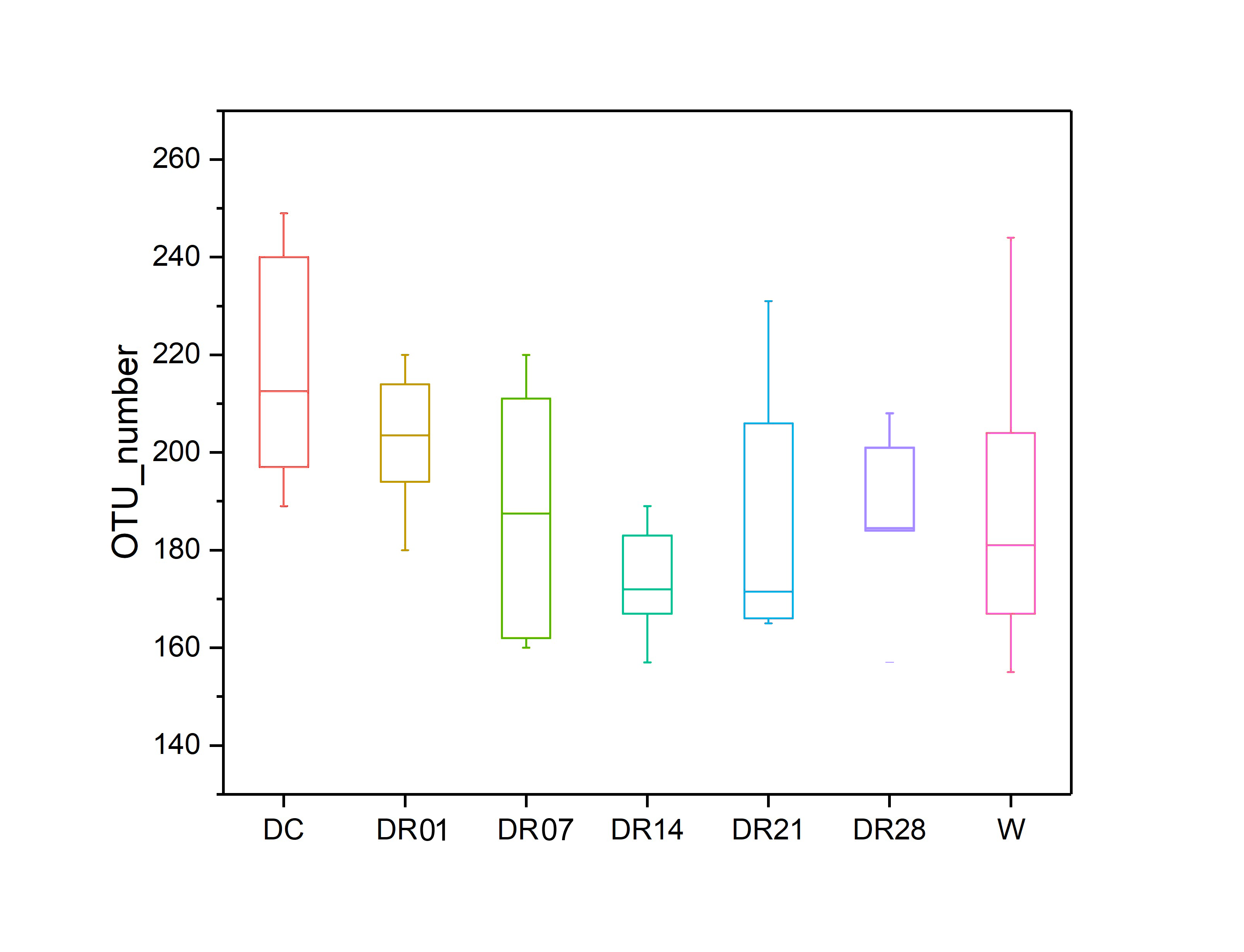

Supplement: Supplementary Figure 3 — Number of operational taxonomic units (OTUs) in different groups. Each box plot represents median, interquartile range, minimum, and maximum values. [file Image_3.TIFF]

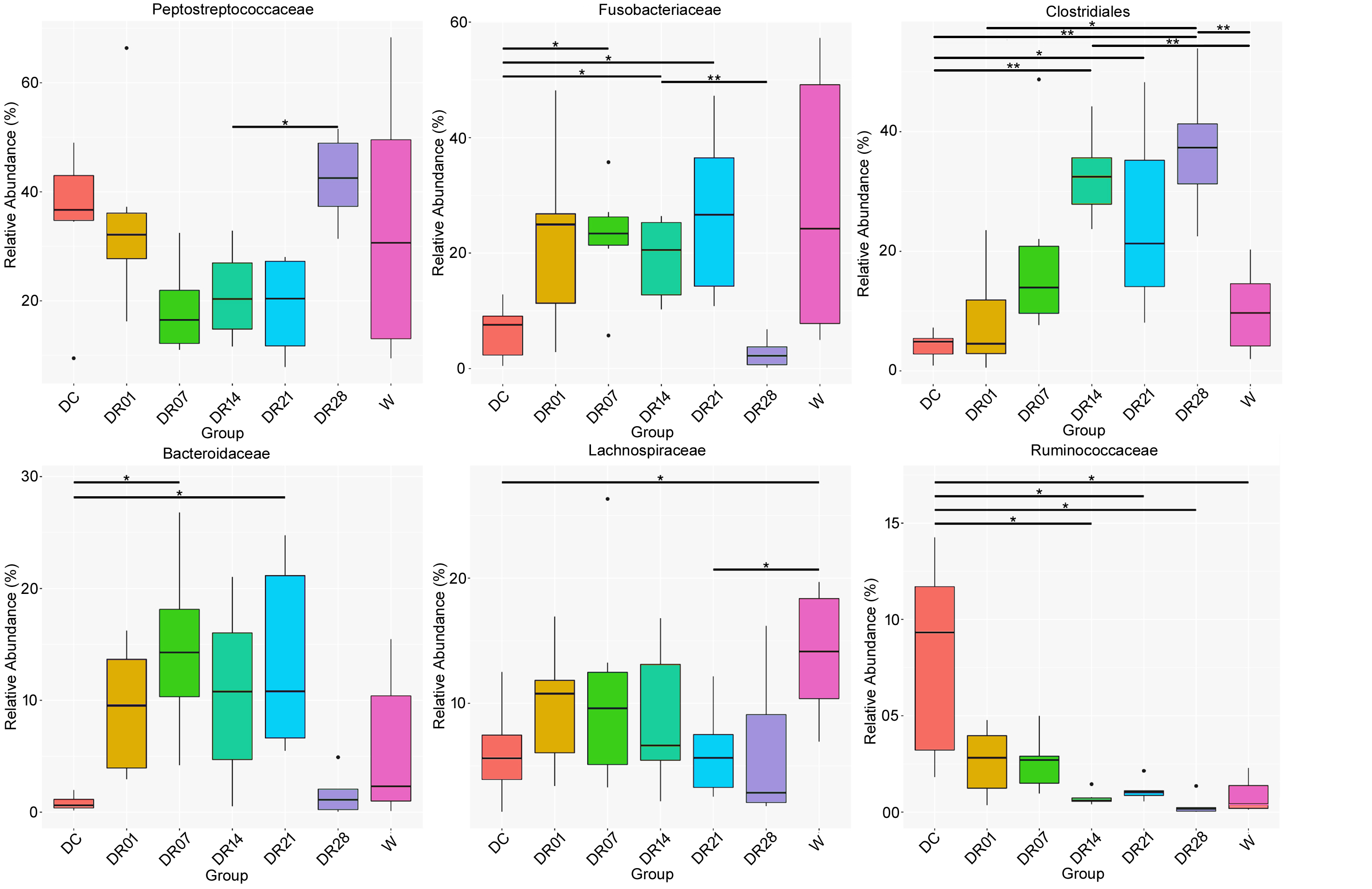

Supplement: Supplementary Figure 4 — The relative abundance of fecal microbiota at family level in feces of raw-fed wolves and dogs on a diet shift from a processed kibble diet to a raw diet (an asterisk means that q < 0.05; a double asterisk means that q < 0.01). [file Image_4.TIF]

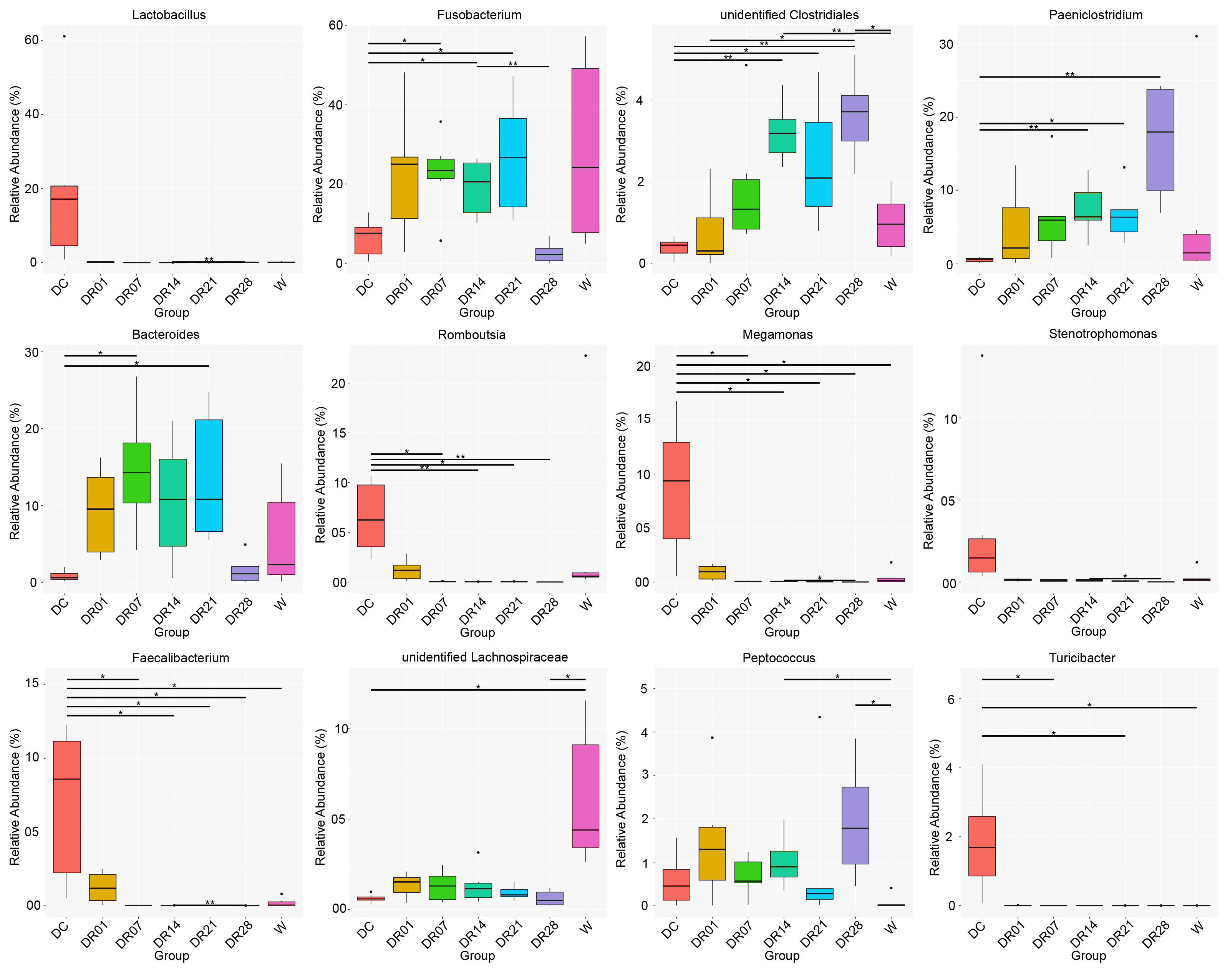

Supplement: Supplementary Figure 5 — The relative abundance of fecal microbiota at genus level in feces of raw-fed wolves and dogs on a diet shift from a processed kibble diet to a raw diet (an asterisk means that q < 0.05; a double asterisk means that q < 0.01). [file Image_5.TIF]

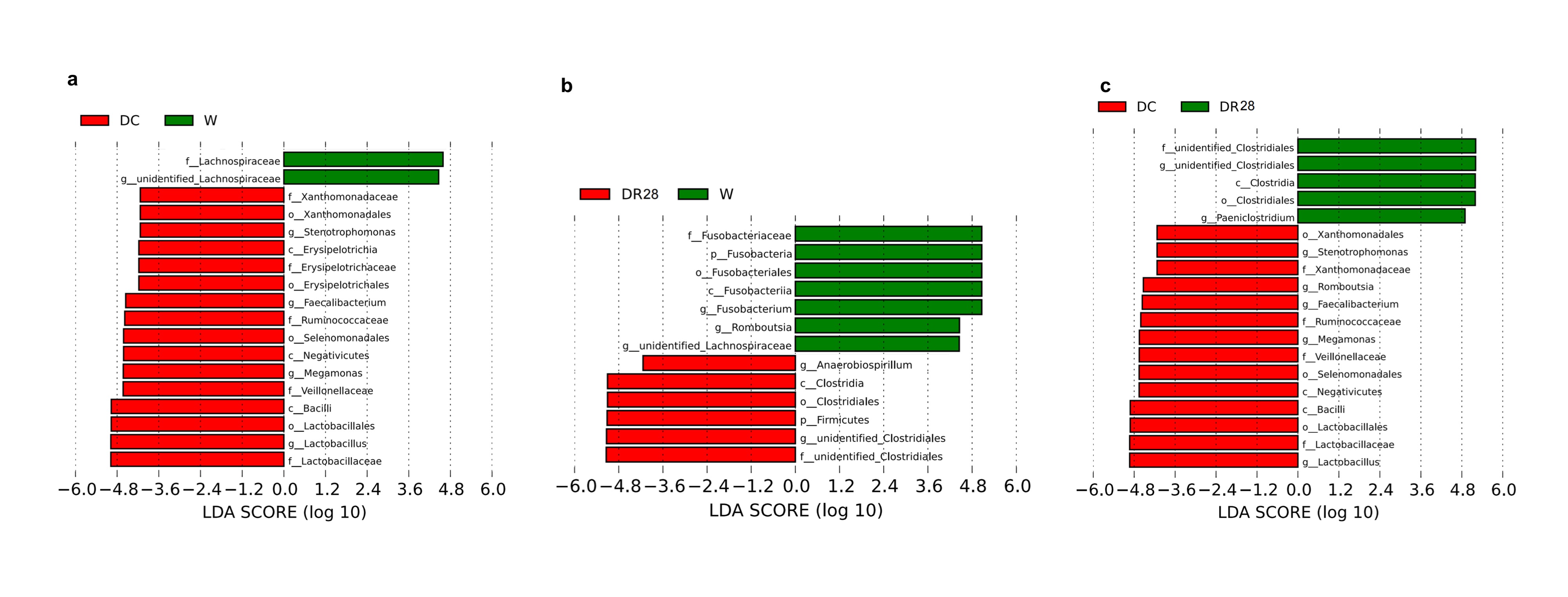

Supplement: Supplementary Figure 6 — Linear discriminant analysis effect size (LEfSe) analysis of 16S rRNA gene sequences. Only LDA values >4 are shown in (A–C). [file Image_6.JPEG]

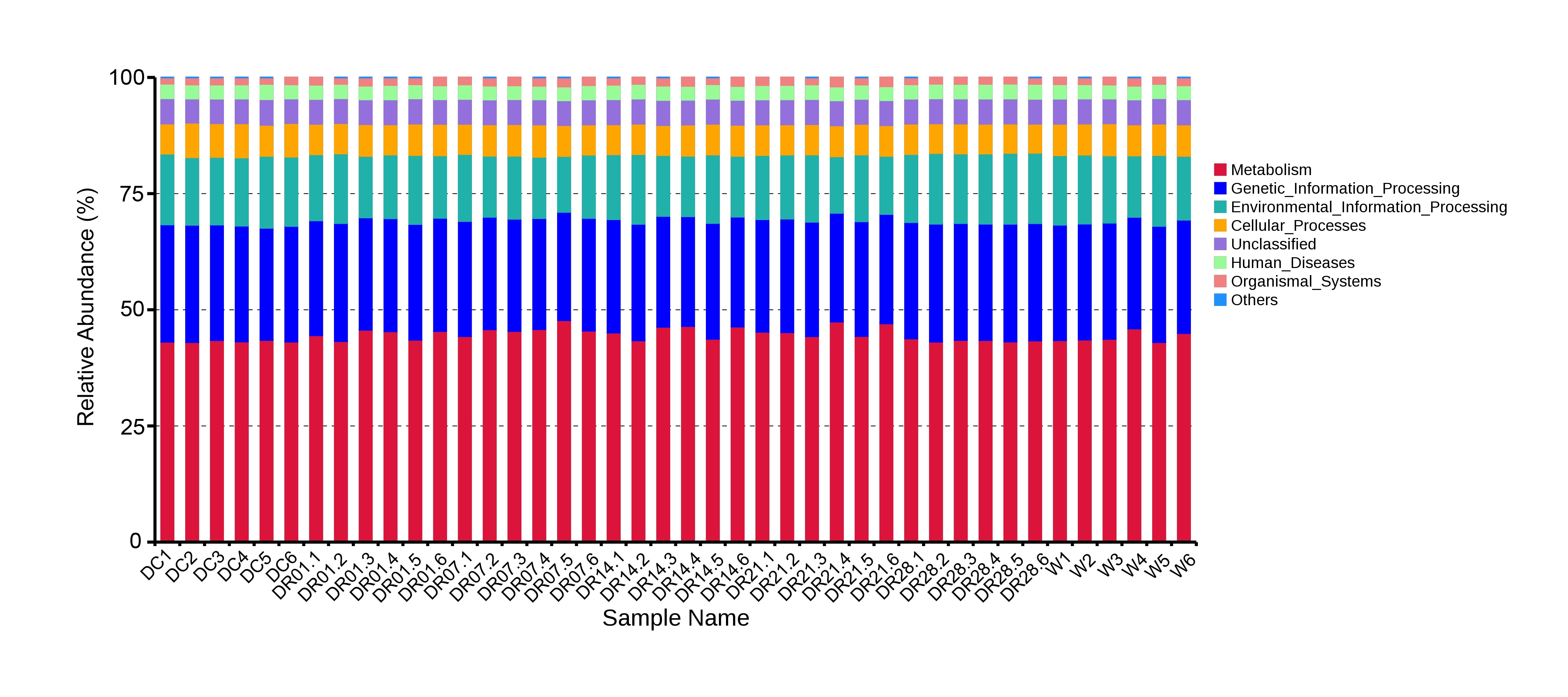

Supplement: Supplementary Figure 7 — Predicted functional composition of metagenomes based on 16S rRNA gene sequencing data. [file Image_7.JPEG]

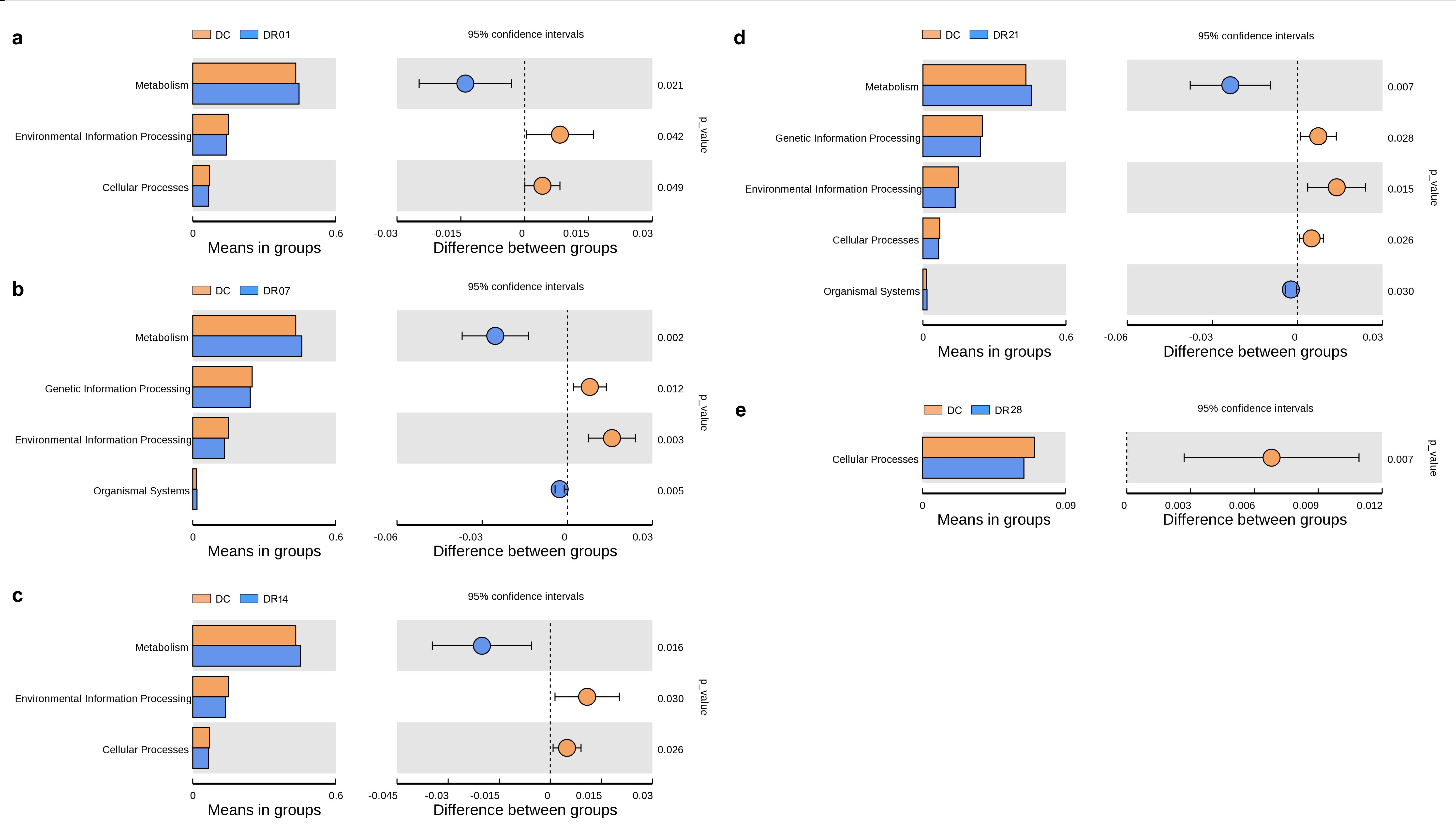

Supplement: Supplementary Figure 8 — The differences of the functional categories at the first level between dogs fed kibble diet and raw diet at different time points. (a) Dogs fed kibble diet vs. dogs fed raw diet for 1 day. (b) Dogs fed kibble diet vs. dogs fed raw diet for 7 days. (c) Dogs fed kibble diet vs. dogs fed raw diet for 14 days. (d) Dogs fed kibble diet vs. dogs fed raw diet for 21 days. (e) Dogs fed kibble diet vs. dogs fed raw diet for 28 days. [file Image_8.JPEG]

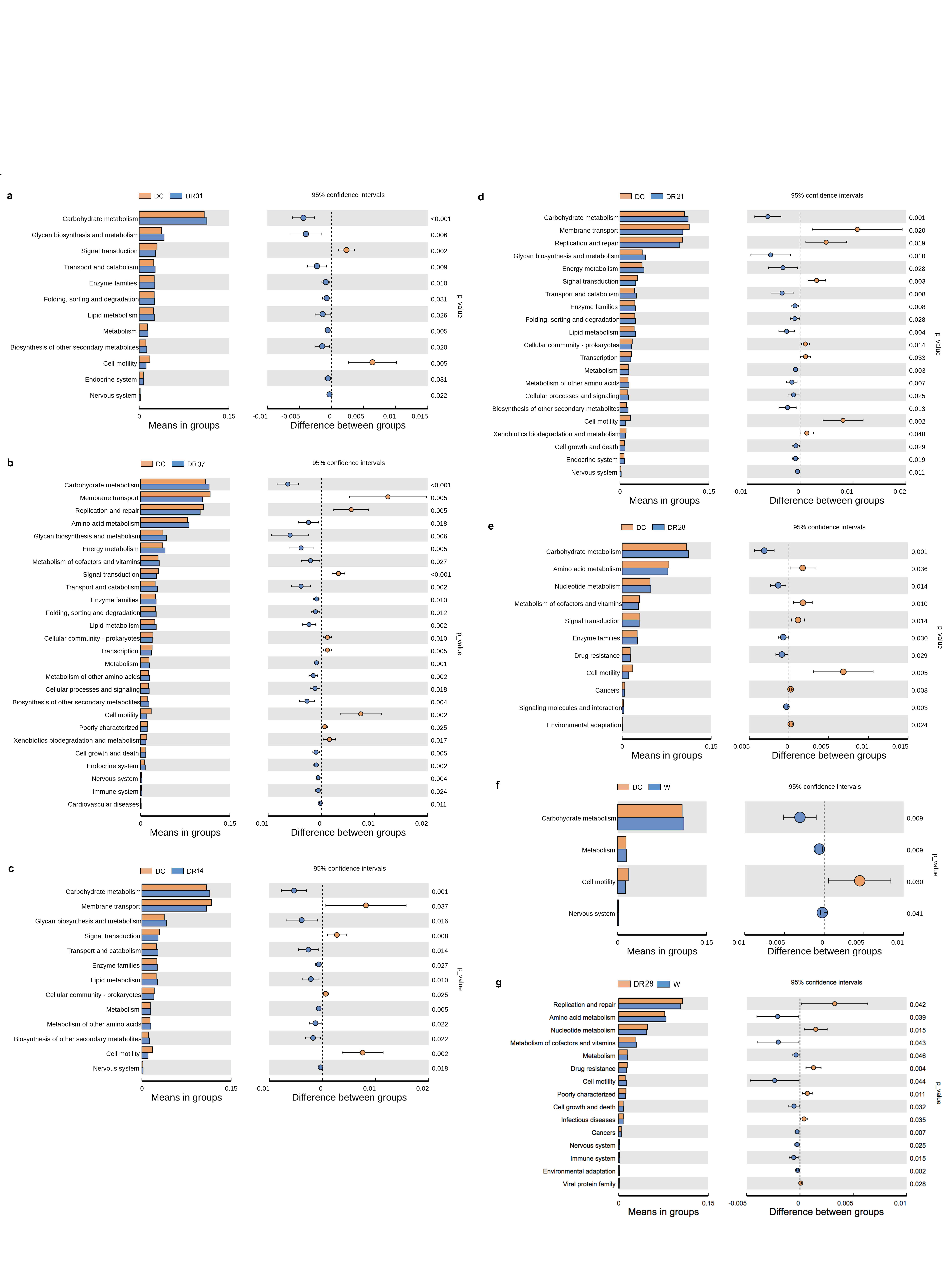

Supplement: Supplementary Figure 9 — The differences of the functional categories at the second level between dogs fed commercial diet and raw diet at different time points. (a) Dogs fed kibble diet vs. dogs fed raw diet for 1 day. (b) Dogs fed kibble diet vs. dogs fed raw diet for 7 days. (c) Dogs fed kibble diet vs. dogs fed raw diet for 14 days. (d) Dogs fed kibble diet vs. dogs fed raw diet for 21 days. (e) Dogs fed kibble diet vs. dogs fed raw diet for 28 days. (f) Dogs fed kibble diet vs. wolves. (g) Dogs fed raw diet for 28 days vs. wolves. [file Image_9.JPEG]

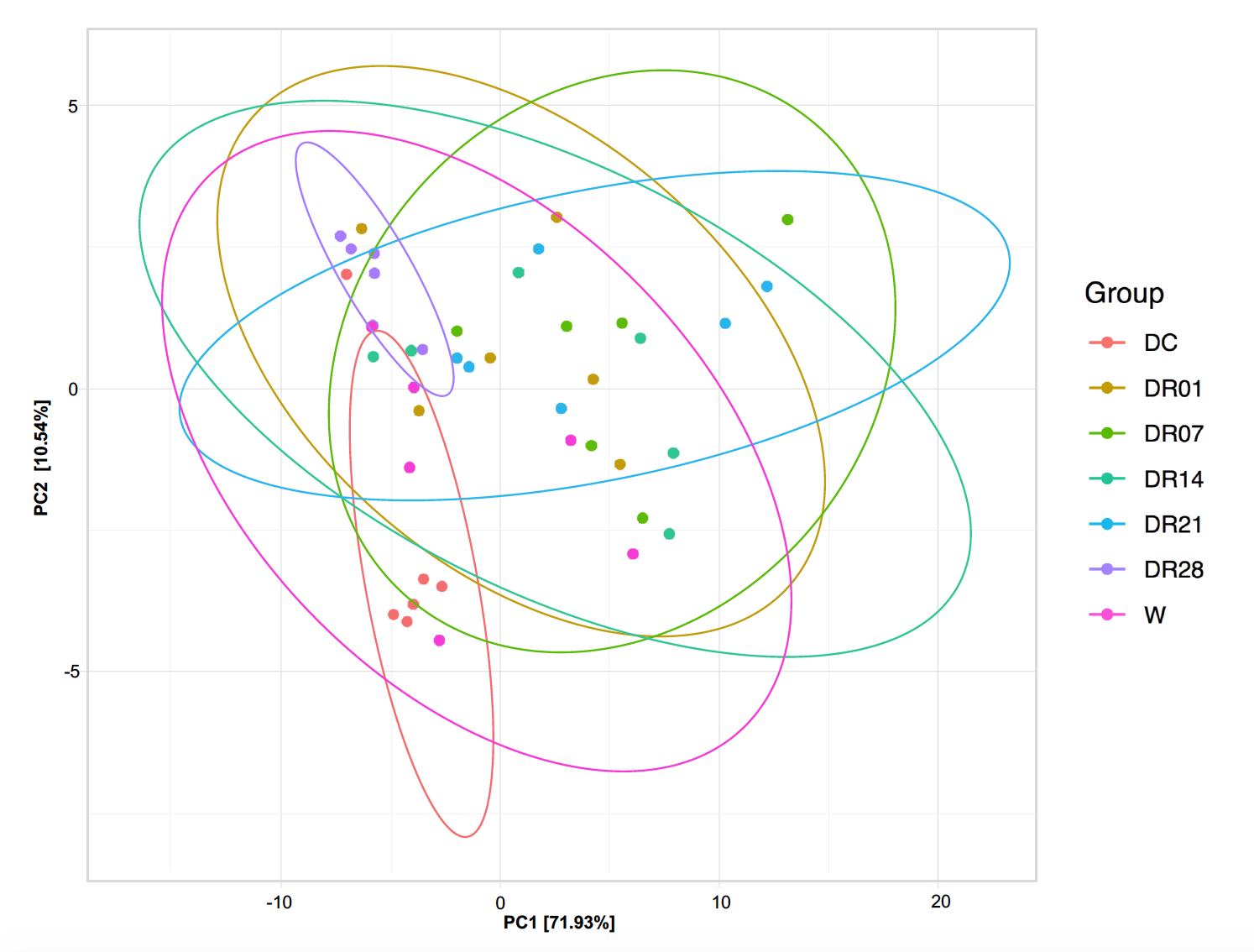

Supplement: Supplementary Figure 10 — Principal component analysis of the predicted gene function in raw-fed wolves and dogs on a diet shift from a processed kibble diet to a raw diet. [file Image_10.PNG]
