# Supplementary material for: The Fecal Microbiota of Dogs Switching to a Raw Diet Only Partially Converges to That of Wolves
Source: Front Microbiol. 2021 Sep 29;12:701439. doi: 10.3389/fmicb.2021.701439 (PMC8511826; doi:10.3389/fmicb.2021.701439)
Supplement: Supplementary file 11 [file Table_1.DOCX]

Supplemental Table 1. Metadata of the animals used in the study

| Animal | Age | Gender |
| --- | --- | --- |
| Dog 1 | 1.5 | M |
| Dog 2 | 2.1 | M |
| Dog 3 | 2.1 | F |
| Dog 4 | 2.1 | F |
| Dog 5 | 2.1 | M |
| Dog 6 | 4.0 | M |
| Wolf 1 | 4.0 | M |
| Wolf 2 | 4.0 | M |
| Wolf 3 | 4.0 | F |
| Wolf 4 | 6.2 | M |
| Wolf 5 | 6.2 | F |
| Wolf 6 | 8.0 | M |
