# Supplementary material for: The Fecal Microbiota of Dogs Switching to a Raw Diet Only Partially Converges to That of Wolves
Source: Front Microbiol. 2021 Sep 29;12:701439. doi: 10.3389/fmicb.2021.701439 (PMC8511826; doi:10.3389/fmicb.2021.701439)
Supplement: Supplementary file 12 [file Table_2.DOCX]

Supplementary Table S2. Chemical composition of the kibble diet used in the study^1^

| Item | Raw diet^1^ | Kibble diet^2^ |
| --- | --- | --- |
| Crude protein (CP) | 14.6% | ≥24.0% |
| Crude fat (EE) | 24.0% | ≥14.0% |
| Crude fiber (CF) | 0.0% | ≤4.0% |
| Ash | 0.7% | ≤10.0% |
| Moisture | 60.7% | ≤10.0% |

^1^ Calculated value based on USDA FoodData Central (https://fdc.nal.usda.gov/). Ingredients: 83% raw chicken meat and bone (CP:14%; EE:27.3%; CF:0%; Ash: 0.64%; moisture: 58.1%) plus 13% kg raw chicken without feathers and organs (CP:17.4%; EE:8.1%; CF:0%; Ash: 1.17%; moisture: 73.2%).

^2^ Ingredients: chicken, beef, corn, wheat, corn gluten, brewer’s yeast, soybean oil, fish oil, egg product, beet pulp.
